# High Levels of IL-10 and CD4+CD25hi+ Treg Cells in Endemic Burkitt’s Lymphoma Patients

**DOI:** 10.3390/biomedicines3030224

**Published:** 2015-08-04

**Authors:** Godfred Futagbi, Ben Gyan, Harriet Nunoo, John K.A. Tetteh, Jennifer E. Welbeck, Lorna Awo Renner, Michael Ofori, Daniel Dodoo, Dominic A. Edoh, Bartholomew D. Akanmori

**Affiliations:** 1 Department of Animal Biology and Conservation Science, College of Basic and Applied Sciences, University of Ghana, Legon, Accra, Ghana; E-Mails: hettydear@yahoo.com (H.N.); adoteiedoh@yahoo.com (D.A.E.); 2 Immunology Department, Noguchi Memorial Institute for Medical Research, College of Health Sciences, University of Ghana, Legon, Accra, Ghana; E-Mails: bgyan@noguchi.mimcom.org (B.G.); jtetteh@noguchi.mimcom.org (J.K.A.T.); mofori@noguchi.mimcom.org (M.O.); ddodoo@noguchi.mimcom.org (D.D.); 3 Department of Child Health, School of Medicine and Dentistry, College of Health Sciences, University of Ghana, Accra, Ghana; E-Mails: jenniferwelbeck20@yahoo.com (J.E.W.); lornarenner@gmail.com (L.A.R.); 4 Division of Immunization, Vaccines & Emergencies (IVE), WHO Regional Office for Africa, Cité Djoué, PO Box 06 Brazzaville, Congo Republic; E-Mail: akanmorib@who.int

**Keywords:** Burkitt’s lymphoma, malaria, EBNA1, Th2, Treg cells

## Abstract

Background: The interplay between Epstein-Barr virus infection, malaria, and endemic Burkitt’s Lymphoma is not well understood. Reports show diminished EBV-specific Th1 responses in children living in malaria endemic areas and deficiency of EBNA1-specific IFN-γ T cell responses in children with endemic Burkitt’s Lymphoma (eBL). This study, therefore, examined some factors involved in the loss of EBNA-1-specific T cell responses in eBL. Methods: T-cell subset frequencies, activation, and IFN-γ- or IL-4-specific responses were analyzed by flow-cytometry. Plasma cytokine levels were measured by ELISA. Results: CD4+ and CD8+ cells in age- and sex-matched healthy controls (*n* = 3) expressed more IFN-γ in response to all immunostimulants than in pediatric endemic BL (eBL) patients (*n* = 4). In healthy controls, IFN-γ expression was higher than IL-4 expression, whereas in eBL patients the expression of IL-4 by CD4+ cells to EBNA-1 was slightly higher than IFN-γ. Moreover, the blood levels of TNF-α was significantly lower (*p* = 0.004) while IL-10 was significantly higher (*p* = 0.038), in eBL patients (*n* = 21) compared to controls (*n* = 16). Additionally, the frequency of CD4+CD25hi+ T cells was higher in both age-matched acute uncomplicated malaria (*n* = 26) and eBL (*n* = 14) patients compared to healthy controls (*n* = 19; *p* = 0.000 and *p* = 0.027, respectively). Conclusion: The data suggest that reduced Th1 response in eBL might be due to increased levels of IL-10 and T reg cells.

## 1. Introduction

Endemic Burkitt’s Lymphoma (eBL) is a common childhood malingnacy in Equatorial Africa [1,2] Strong and consistent association of BL and Epstein-Barr virus (EBV) is known, and EBV and malaria are believed to be co-factors in the development of eBL [3,4,5]. EBV is also associated with some malignancies, including nasopharyngeal carcinoma and AIDS-related non-Hodgkin lymphoma. [6,7,8] Expression of latent and lytic genes by EBV does not only differ from one malignancy to another but also from one stage to another. However, Epstein-Barr nuclear antigen 1 (EBNA-1), which is a latent protein, is expressed in all EBV-associated lymphomas. Furthermore, it has been reported that some of the EBV infected B lymphocytes in blood express only EBNA-1 [9], making it an important target.

Aside the speculations that malaria could play a role in early events such as providing an additive risk for development of B-cell clones with chromosome translocations associated with the tumor [10], there is also the need to understand the immunological mechanisms by which infection with *Plasmodium falciparum* may contribute to eBL. This is moreso since there is no conclusive explanation for the fact that about ninety percent (90%) of the world population is latently and permanently infected with EBV [11] and yet Burkitt’s lymphoma is predominantly associated with malaria endemic areas [12], at least before the advent of HIV and AIDS. It is easy to guess that during *P. falciparum* malaria the immune surveillance may be derailed as a result of imbalances in the immune regulation.

Because BL cells lack the ability to process EBNA-1 onto MHC Class I molecules, it was initially assumed that CD8+ cells do not recognize EBNA-1. It has now been shown that EBNA-1 can be presented on MHC Class I molecules if it is exogenously processed and EBNA-1-specific CD8+ cytotoxic T lymphocytes (CTLs) that do recognize EBV-transformed cells have been identified [13,14]. EBNA-1 can also be processed onto MHC class II for CD4+ T cells and in both *in vitro* system and a mouse model EBNA-1-specific CD4+ T cells have been shown to have the capacity to prevent BL development [15,16,17,18]. Furthermore, among the virus-encoded antigens that stimulate CD4+ T cells, EBNA-1 is preferentially recognized. However, it has also been found that there is diminished EBV-specific Th1 responses in children living in malaria-holoendemic areas [19] and deficiency of EBNA-1-specific IFN-γ T cell responses in children with eBL [8]. The mechanism is not clear and in this study we examined some of the possible factors that may contribute to the deficiency of EBNA-1-specific Th1 responses.

## 2. Experimental Section

### 2.1. Study Population and Inclusion Criteria

This was a case control study involving two categories of patients and a third group of age-and sex-matched healthy controls. Twenty-one children with endemic Burkitt’s lymphoma (eBL), aged from three to 11 years, were enrolled at the Korle-Bu Teaching Hospital. Recruitment followed clinical and confirmatory diagnosis. BL patients who had started treatment with malaria or asymptomatic parasitemia were excluded from the study. Twenty-six children with acute uncomplicated malaria, confirmed on blood film, of ages ranging from three to 14 years were also enrolled from University of Ghana hospital and the Ghana Atomic Energy Commission clinic. Nineteen age-matched healthy controls without parasitaemia were also enrolled for the study (Table 1). Informed consent was obtained from parents or guardians of children before enrollment in the study. The study was conducted in accordance with the Declaration of Helsinki. The Institutional Review Board at Noguchi Memorial Institute granted ethical approvals for the study (Certified Protocol Number (CPN): 047/07-08).

**Table 1:** Clinical characteristics of the study participants.

Characteristic	Participants			Significance		
	Malaria Patients (MAL)	eBL Patients	Controls (CON)	CON *vs.* MAL	CON *vs.* eBL	eBL *vs.* MAL
	Mean (95% CI)			*p*-Value		
Male ^#^	14	14	13	-	-	-
Female ^#^	12	7	6	-	-	-
Mean Age (range, years)	8.6 (3 to 14)	6.9 (3 to 11)	8.7 (5 to 14)	-	-	-
WBC ^#^	8.75	8.17	5.95	0.0039	0.0017	0.5518
(×10^3^/μL)	(7.36 to 10.15)	(6.96 to 9.38)	(5.43 to 6.47)			
HGB (g/dL)	11.63	10.78	12.10	-	0.2906 *	-
	(10.9 to 12.3)	(7.7 to 13.9)	(11.5 to 12.7)			
Parasitemia (parasites/μL)	475	-	-	-	-	-
	(76 to 9275)					

^#^ represents number.* *p*-value for student’s *t*-test.

### 2.2. Sample Collection and Processing

Blood was collected into sterile heparin and EDTA tubes (BD Vacutainer™) using sterile safety-lok™ blood collection set and were processed within three hours. Peripheral blood mononuclear cells (PBMC) were isolated by Ficoll-Histopaque (Sigma-Aldrich, St. Louis, MO, USA) density gradient centrifugation using a 10ml cell-separator tube (LeucoSep™ tube; Sigma-Aldrich). PBMC layer was harvested, washed three times in RPMI1640 containing 10% heat-inactivated foetal calf serum (FCS), and supplemented with penicillin/streptomycin and l-glutamine. The PBMC were then dispersed in a cold freezing mix (10% DMSO in FCS), aliquoted into cryotubes, placed in Mr. Frosty® (Nalgene cryo1 °C freezing container, Nalgene, Rochester, NY, USA) and frozen at −80 °C overnight. The cells were transferred into liquid nitrogen the following day for cryopreservation. The plasma was stored in vials at −80 °C.

### 2.3. Parasitological and Haematological Examinations

An automated haematology analyzer (Sysmex KX-21, Sysmex, Kobe, Japan) was used to determine all the haematological parameters of the participants. The absolute counts of lymphocytes were determined from this analysis. All the venous blood samples were examined for presence of parasite-infected red blood cells to confirm infection with *P. falciparum* and also to exclude asymptomatic healthy donors. Thick and thin blood smears were prepared, dried and the thin smears fixed in methanol. The films were then stained with freshly prepared 10% Giemsa (BDH Laboratory Supplies, Poole BH15 ITD, Leics., UK) and washed cautiously and thoroughly under running tap water. The slides were dried and observed with immersion oil under a light microscope (Olympus BH-2, Olympus Optical Co., Tokyo, Japan) at 100× magnification, for the presence of *P. falciparum* infected red blood cells.

### 2.4. Cell Phenotyping

PBMC were quickly thawed in a water bath at 37 °C and washed twice in RPMI1640 containing 10% heat-inactivated FCS, supplemented with penicillin/streptomycin and l-glutamine. The cells were then stained with Trypan blue to ascertain cell viability and viable cell concentration adjusted to 1 × 10^6^/mL in a staining buffer, and stained with combinations of T-cell subset or activation marker, CD25 or CD95-specific monoclonal antibodies conjugated to fluorescein isothiocyanate (FITC), phycoerythrin (PE) or PE-Cy5. Surface staining was done with antibodies directed against CD3 (HIT3a; BioLegend, San Diego, CA, USA), CD4 (RPA-T4; BioLegend), CD8 (RPA-T8; BioLegend), CD25 (BC96; BioLegend), and CD95 (DX2; BioLegend).

Three microliters (3 µL) of the antibodies were added to the cells and mixed. Stained PBMC were incubated at room temperature in the dark for 15 min. After incubation, the cells were washed with FACS Buffer three times with supernatants decanted. Cells were re-suspended in 200 µL of FACS buffer for acquisition or re-suspended in 1 mL of fixation/permeabilization buffer and taken through intracellular staining.

### 2.5. Intracellular Cytokine Detection

PBMC were first stimulated with antigens or mitogens of interest before any staining was done, as described below. Before intracellular staining, the cells were stained with antibodies directed against CD4 (RPA-T4; BioLegend), CD8 (RPA-T8; BioLegend), and CD25 (BC96; BioLegend) as described above. Intracellular staining was done using FoxP3 staining buffer set (cat 00-5523, eBiosciences, San Diego, CA, USA), according to the manufacturer’s instructions. However, the data on FoxP3+ cells were not presented in this report.

Surface-labelled PBMC were fixed and permeabilized by adding 1ml of freshly prepared fixation/permeabilization buffer and incubated in the dark at room temperature for 20 min. After incubation, the cells were washed twice with 2 mL of 1× permeabilization wash buffer and supernatant carefully aspirated each time. The fixed/permeabilized cells were re-suspended in residual permeabilization wash buffer. The 3 µL of antibodies for intracellular staining; interferon-gamma (IFN-γ, 4S.B3; BioLegend), interleukin-4 (IL-4, MP4-25D2; BioLegend) and FoxP3 (PCH101, eBiosciences), were then added to the cells, pulse vortexed and incubated in the dark for 20 min. After incubation, the PBMC were first washed with 2 mL of 1× permeabilization buffer and, second, with 2 mL of flow cytometry staining buffer. The stained cells were then re-suspended in 200 µL of flow cytometry staining buffer for acquisition.

### 2.6. In Vitro Stimulation

PBMC were cultured for 6 h in the presence of stimulants, such as PepTivator-EBV EBNA-1 (EBNA-1; Miltenyi Biotec, Auburn, CA, USA) peptide pool and phytohaemagglutinin (PHA), at 1.5 × 10^6^ PBMC/well in 150 µL medium in 96 flat-bottom plates (culture medium: RPMI supplemented with penicillin/streptomycin and 10% human pool AB serum). After 2 h, 1:1000 brefeldin A was added to allow accumulation of cytokines in the cytosol and incubated for the remainder of the 6 h. Six hours was chosen for the cytokine secretion assay with respect to the EBNA-1 manufacturer’s recommendations (Miltenyi Biotec). 3 mL of EBNA-1 peptide pool of stock concentration of 30 nmol of each peptide per mL was added to each well making a final concentration approximately 0.6 nmol/mL (or 1 µg/mL) of each peptide. The pool of EBNA-1 peptides consists mainly of 15-mer sequences with 11 amino acids overlap, covering the compete sequence (except the GA region) of the EBNA-1 (Miltenyi Biotec). Therefore, the peptides spanned all the regions (including the entire C-terminal region) of the protein, encompassing all possible epitopes. PHA was added as a positive control at a final concentration of 5 µg/mL. Medium without stimulant was included as a negative control.

After stimulation, the cells were carefully collected by pipetting up and down and wells rinsed with buffer. The cells were then washed in FACS buffer and aliquoted at a minimum of 100,000 cells in 100 µL for surface and intracellular staining as described above.

### 2.7. Flow Cytometric Acquisition and Analyses

Flow cytometry acquisition was done using a FACScan flow cytometer (Becton Dickinson, San Jose, CA, USA). Two or three-color flow cytometry analyses panels were employed. Appropriate isotype controls were also analyzed. Before data acquisition, instrument parameters were checked and optimized using CaliBRITE beads (Becton Dickinson). Data were acquired with Multiset CellQuest software (Becton Dickinson) and analyzed using FlowJo software (Treestar, Ashland, OR, USA). Lymphocyte population was set using FSC display and gated.

### 2.8. Measurement of Cytokines by ELISA

Levels of the cytokines, tumor necrosis factor-alpha (TNF-α), and Interleukin-10 (IL-10) were determined in plasma of eBL patients as well as their healthy counterparts by ELISA. Briefly, microtiter plates were coated with 50 μL/well of purified anti-human TNF-α or anti-human IL-10 monoclonal antibody at 2 μg/mL and incubated overnight at 4 °C. After washing and blocking, standard recombinant human TNF-α or IL-10 was added at serial dilutions from 200 pg/mL to 3 pg/mL in addition to undiluted plasma at 50 μL/well in duplicates. After incubation and washing, a biotinylated anti-human TNF-α or IL-10 was added and the plates again incubated and washed. An avidin peroxidase conjugate was then added at 2.5 μg/mL followed by another incubation. After washing, OPD substrate was added and the plates developed, and read at 492 nm. The optical density values of the standards were used to draw the appropriate curves that were used to transform the sample optical density values to concentrations in pg/mL.

### 2.9. Statistical Analyses

Data was analyzed using SPSS 16.0 (SPSS Inc., Chicago, IL, USA) and GraphPad Prism (GraphPad Prism, GraphPad Software, San Diego, CA, USA) software. Statistical analyses were performed using Student t-test and Kruskall-Wallis or Mann–Whitney rank sum nonparametric tests. *p* < 0.05 was considered significant.

## 3. Results

### 3.1. Characteristics of Study Participants

Participants recruited for this study included; 19 healthy controls, 21 eBL patients and 26 malaria patients, with mean ages 8.7 (range: 5–14), 6.9 (range: 3–11) and 8.6 (range: 3–14) years respectively. Absolute number of WBC, were significantly higher in malaria patients and eBL patients compared to healthy controls (*p* = 0.004, *p* = 0.002, respectively**)**. There were no differences between malaria and eBL patients with regard to absolute numbers of WBC (*p* = 0.552; Table 1).

### 3.2. Decrease in Responses by T Cells of eBL Patients

**Figure 1:**
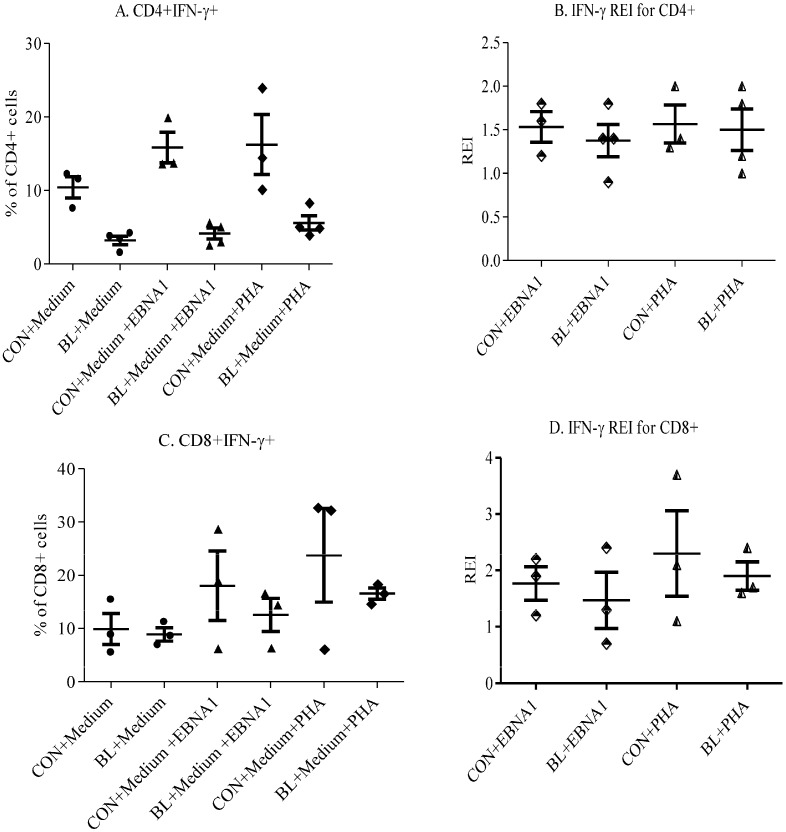
IFN-γ Production by CD4+ and CD8+ T Cells in Response to EBNA1. IFN-γ expression by CD4+ cells (**A**) and CD8+ cells (**C**) compared between BL patients and controls. The respective REIs are shown in (**B**) and (**D**). REI = percentage of cells of interest expressing IFN-γ after culture with a stimulus (EBNA1 or PHA) divided by percentage of the same cells expressing IFN-γ after culture in medium without stimulus. Bars show mean ± SE.

**Figure 2:**
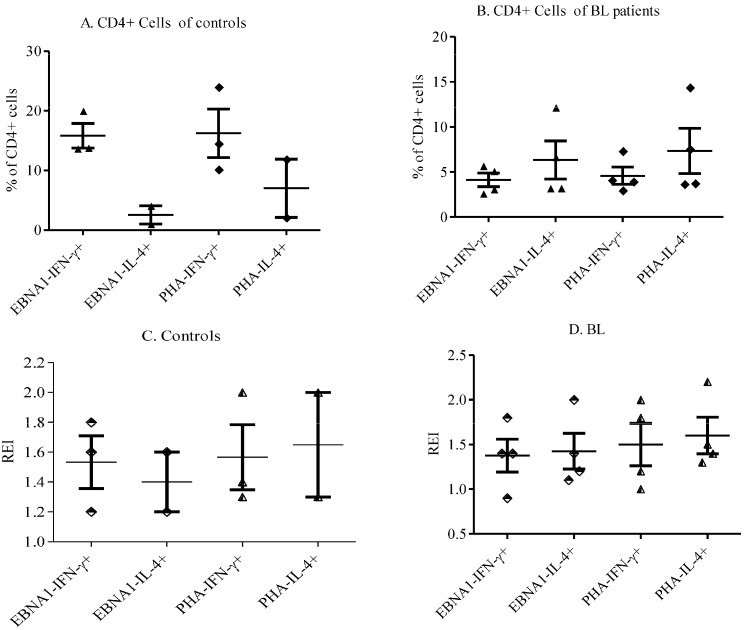
IFN-γ and IL-4 Expression to EBNA1 and PHA by CD4+ Cells. Comparisons were made between IFN-γ and IL-4 expression by CD4+ of controls (**A**) and eBL patients (**B**). REIs for IFN-γ and IL-4 for controls and eBL patients are also presented in the lower panels (**C** and **D**, respectively). Bars show mean ± SE.

To evaluate the specific responses of T cells, PBMC were stimulated with EBNA-1. PHA and medium without any stimulation were used as positive and negative controls, respectively. Only three controls and four eBL patients were included in the intracellular staining, due to limited resources. They were selected based on age- and sex-matching and being used in other assays. Figure 1 shows the response of CD4+ and CD8+ T cells to EBNA-1. Generally, CD4+ and CD8+ cells expressed more IFN-γ in controls than in eBL patients to all the stimulants (Figure 1A,C). It was also observed that CD4+ T cells from healthy controls had higher interferon-γ relative expression index (IFN-γ REI) to EBNA-1 compared to those from eBL patients (Figure 2B). This indicates a decrease in EBNA-1-specific response by CD4+ T cells in eBL. On the contrary, CD8+ cells of patients and controls had similar IFN-γ REI (Figure 1D). This indicates that, although there is general decrease in response by CD8+ cells, there is no reduction in EBNA-1-specific Th1 response by CD8+ T cells in eBL.

### 3.3. Th1/Th2 Responses to EBNA-1 in eBL Patients

To identify any imbalance in Th1/Th2 immune response dichotomy, we analyzed IFN-γ and IL-4 expression by CD4+ T cells in response to EBNA-1 stimulation in patients and controls. The frequency of IFN-γ expression was higher than IL-4 expression to all stimulants in healthy controls (Figure 2A). However, in eBL, the expression of IL-4 by CD4+ cells to EBNA-1 and PHA was slightly higher than IFN-γ (Figure 2B). IFN-γ REI of CD4+ cells to EBNA-1 was higher than IL-4 REI in healthy controls. In eBL patients, on the contrary, IFN-γ and IL-4 REIs of CD4+ cells were similar (Figure 2C,D).

### 3.4. High Plasma Level of TNF-α and IL-10 in eBL Patients

**Figure 3:**
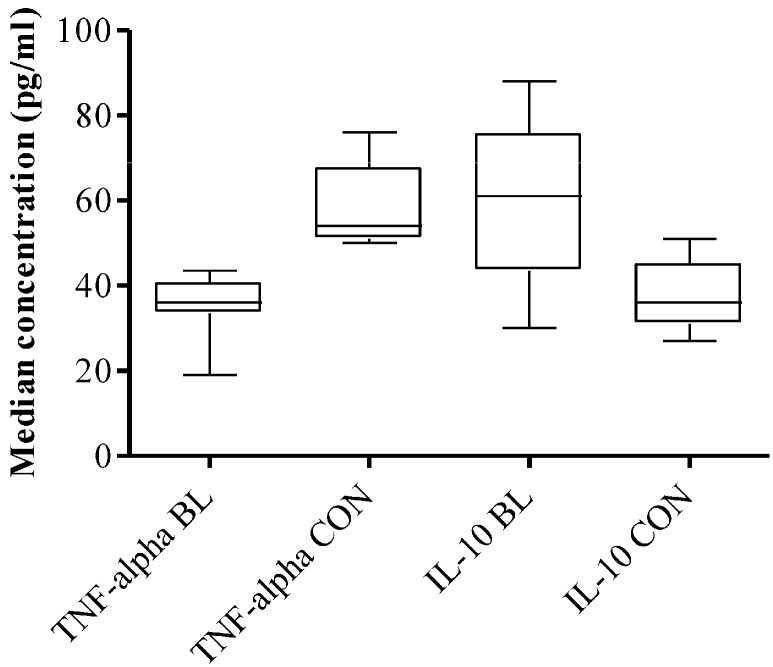
Plasma levels of TNF-alpha and IL-10 of eBL patients (*n* = 21) and controls (*n* = 16) compared. The whiskers show maximum and minimum values.

Plasma levels of TNF-α and IL-10 were also measured to examine Th1/Th2 cytokine balance *in vivo*. The median level of peripheral blood TNF-α was significantly lower in eBL patients compared to healthy controls (*p* = 0.004). Conversely, plasma level of IL-10 was significantly higher in eBL patients than in healthy controls (*p* = 0.038; Figure 3).

### 3.5. High Frequency of CD4+CD25hi+ Treg Cells in Patients

**Figure 4:**
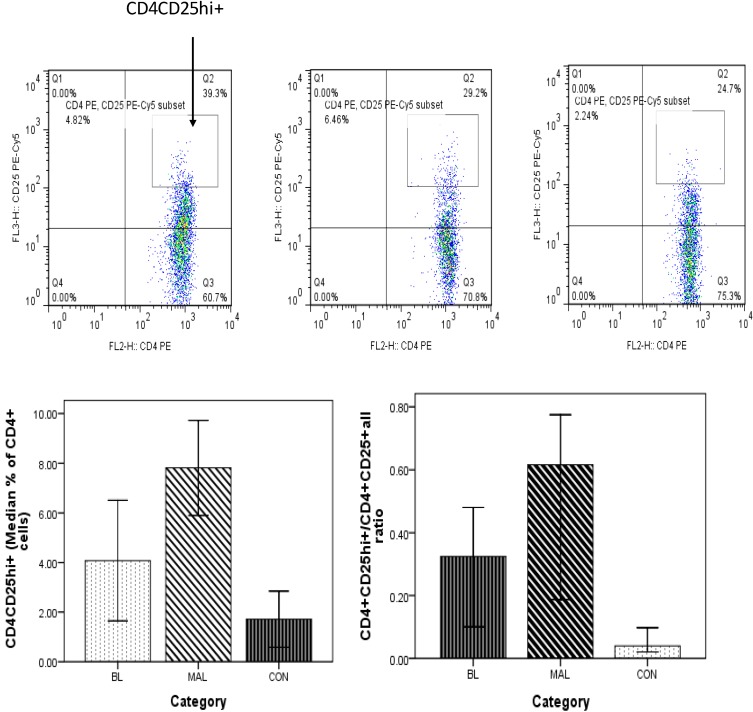
CD25hi expression by CD4+ cells. The quadrants represent CD25 expression by CD4+ cells in eBL (left panels, *n* = 14), acute uncomplicated malaria (middle panels, *n* = 26) and healthy controls (right panels, *n* = 19). The charts compare CD25hi expression by CD4+ cells and CD4+CD25hi+/CD4+CD25+ among the categories. Error bars represent 95% CI.

The frequency of Treg cells was also analyzed to determine their possible role in diminished responses in the eBL patients. The median frequency of CD4+CD25hi+ Treg cells were higher in both malaria and eBL patients compared to healthy controls (*p* = 0.000 and *p* = 0.027, respectively). Data on the frequency of CD4+CD25hi+FoxP3+ Treg cells has yet to be concluded. Additionally, the CD4+CD25hi+/CD4+CD25+ ratio was also examined and found to be significantly higher in malaria and eBL patients compared to the healthy controls (*p* = 0.004 and *p* = 0.014, respectively). No significant differences were found between malaria and eBL patients with regard to the frequency of CD4+CD25hi+ cells and CD4+CD25hi+/CD4+CD25+ ratio (*p* = 0.411 and *p* = 0.148, respectively; Figure 4).

## 4. Discussion

Children living in malaria endemic regions are at the highest risk of having eBL. It has been established that malaria contributes to the development of eBL and it is believed that persistent malaria and associated dysregulation of the immune system could play an important role in the development of eBL. Recent studies have shown diminished EBV-specific Th1 responses in children living in malaria-holoendemic areas [19] and deficiency of EBNA-1-specific IFN-γ T cell responses in children with eBL [8]. This study aimed at shedding light on some of the mechanisms that could lead to reduced specific responses to EBNA-1 and the contribution of malaria infection.

The higher IFN-γ REI for CD4+ T cells from healthy donors compared to those from eBL patients corroborate the loss of EBNA-1-specific response by CD4+T cells in eBL reported in a recent study [8]. The similarity in IFN-γ REI for CD8+ T cells between eBL patients and healthy controls suggests that there is no reduction in EBNA-1-specific Th1 response by CD8+ T cells in eBL. This finding is also in agreement with what has been observed by others [8]. Moreover, in CD4+ T cells, the frequency of IFN-γ expression to all stimulants was higher in healthy controls than in eBL patients. Furthermore, in CD8+ cells, IFN-γ expression to all stimulants was slightly lower in eBL patients compared to the healthy controls. This implies that IFN-γ expression by CD4+ T cells is somehow affected in eBL patients.

To explain this we examined Th1/Th2 dichotomy comparisons that were made between IFN-γ and IL-4 expression by CD4+ cells of controls and eBL patients. The results point to a slight tilting of EBNA-1-specific responses in favor of Th2 in eBL, compared to strongly skewed responses in favor of Th1 in controls. However, further work is required. Th2-weighted responses are known to suppress T-cell function [20]. The low plasma level of TNF-α and the high level of IL-10 in patients is an indication of similar dysregulation of the immune response *in vivo*. IL-10 promotes Th2 responses while down-regulating Th1 responses, particularly proliferation and activation of CTLs. Priming of EBNA-1-specific CD4+ T cells by dendritic cells might also be impaired *in vivo* by IL-10 [21]. TNF-α, as its name connotes, has the ability to kill tumor cells. It has been shown in an islet cancer of the pancreas that TNF, as well as IFN-γ, can, at least, drive cancer cells into senescence [22,23]. Down-regulation of TNF-α production in eBL patients indicates that the anti-tumor mechanism involving TNF-α is rather reduced in patients where it is most needed.

The possible involvement of Treg cells in eBL development was also examined. Treg cells are involved in immunological tolerance and malaria is known to promote the development and function of Treg cells. In a murine model, addition of antigen-specific Treg cells inhibits effector T cell response [24]. Our data show a significant increase in the frequency of CD4+CD25hi+ cells in both malaria and eBL. This suggests the involvement of Treg cells in immunity to eBL and may support the speculation that malaria could contribute to the development of EBV+ tumors through activities of Treg cells. Previous studies have pointed to Treg cells in the development of tumors [25,26] but this is the first time they are implicated in eBL. CD4+CD25hi+ Treg cells is a composite group, some express FoxP3 and exert their suppressive activity through cell-cell contact and others lack the FoxP3 (Tr1 cells) and exert their activity through secretion of immunosuppressive cytokines such as TGF-β and IL-10 [27,28]. Tr1 cells are known to secrete IL-10 [29] and this suggests that CD4+CD25hi+ cells may contribute to the high IL-10 levels in the patients. EBV-infected B cells also produced human IL-10 [30]. CD4+CD25hi+/CD4+CD25+ ratio, which represents Treg cell/effector T cell ratios, was found to be higher in both malaria and eBL patients than in controls. This is an indication of impaired function of effector T cells in the patients. However, further analysis of the CD4+CD25hi+ Treg cells is required to fully understand their role in eBL development.

The high plasma level of IL-10 in eBL, and the high frequency of CD4+CD25hi+ Treg cells in malaria and eBL, give credence to the parallel between dysregulation of the immune system in the two diseases.

## 5. Conclusions

In summary, the data suggest that the reduced Th1 responses in eBL might be due to increased levels of IL-10 and Treg cells. It also indicates that malaria may also contribute to the development of eBL through increased secretion of IL-10 and generation of Treg cells.
